# Use of a targeted, combinatorial next-generation sequencing approach for the study of bicuspid aortic valve

**DOI:** 10.1186/1755-8794-7-56

**Published:** 2014-09-26

**Authors:** Elizabeth M Bonachea, Gloria Zender, Peter White, Don Corsmeier, David Newsom, Sara Fitzgerald-Butt, Vidu Garg, Kim L McBride

**Affiliations:** 1 Department of Pediatrics, The Ohio State University, Columbus, OH, USA; 2 Center for Cardiovascular and Pulmonary Research and The Heart Center, Nationwide Children’s Hospital, 700 Children’s Drive Room WB4275, Columbus, Ohio 43205, USA; 3 Biomedical Genomics Core. The Research Institute at Nationwide Children’s Hospital, Columbus, OH, USA; 4 Department of Molecular Genetics, The Ohio State University, Columbus, OH, USA

**Keywords:** Bicuspid aortic valve, Genetics, Next-generation sequencing, Targeted capture, Combinatorial pooling

## Abstract

**Background:**

Bicuspid aortic valve (BAV) is the most common type of congenital heart disease with a population prevalence of 1-2%. While BAV is known to be highly heritable, mutations in single genes (such as *GATA5* and *NOTCH1*) have been reported in few human BAV cases. Traditional gene sequencing methods are time and labor intensive, while next-generation high throughput sequencing remains costly for large patient cohorts and requires extensive bioinformatics processing. Here we describe an approach to targeted multi-gene sequencing with combinatorial pooling of samples from BAV patients.

**Methods:**

We studied a previously described cohort of 78 unrelated subjects with echocardiogram-identified BAV. Subjects were identified as having isolated BAV or BAV associated with coarctation of aorta (BAV-CoA). BAV cusp fusion morphology was defined as right-left cusp fusion, right non-coronary cusp fusion, or left non-coronary cusp fusion. Samples were combined into 19 pools using a uniquely overlapping combinatorial design; a given mutation could be attributed to a single individual on the basis of which pools contained the mutation. A custom gene capture of 97 candidate genes was sequenced on the Illumina HiSeq 2000. Multistep bioinformatics processing was performed for base calling, variant identification, and *in-silico* analysis of putative disease-causing variants.

**Results:**

Targeted capture identified 42 rare, non-synonymous, exonic variants involving 35 of the 97 candidate genes. Among these variants, *in-silico* analysis classified 33 of these variants as putative disease-causing changes. Sanger sequencing confirmed thirty-one of these variants, found among 16 individuals. There were no significant differences in variant burden among BAV fusion phenotypes or isolated BAV versus BAV-CoA. Pathway analysis suggests a role for the WNT signaling pathway in human BAV.

**Conclusion:**

We successfully developed a pooling and targeted capture strategy that enabled rapid and cost effective next generation sequencing of target genes in a large patient cohort. This approach identified a large number of putative disease-causing variants in a cohort of patients with BAV, including variants in 26 genes not previously associated with human BAV. The data suggest that BAV heritability is complex and polygenic. Our pooling approach saved over $39,350 compared to an unpooled, targeted capture sequencing strategy.

## Background

Congenital bicuspid aortic valve (BAV) is the most common type of cardiac malformation, with an estimated prevalence of 1-2% in the general population [1]. BAV, in which two of the three normal aortic cusps are fused together, encompasses a wide spectrum of clinical phenotypes. The valve abnormality may be isolated in some cases, whereas in others the aortic valve abnormality is present in conjunction with other cardiac malformations [2]. BAV may also be associated with varying degrees of aortic valve stenosis and/or insufficiency as well as with aortopathy. Among BAV patients, there is variability in cusp fusion phenotypes. Right coronary and left coronary (R-L) cusp fusion is more common than right coronary and non-coronary (R-NC) cusp fusion. Moreover, R-L cusp fusion is more often associated with additional cardiac malformations, whereas R-NC cusp fusion is more likely to be associated with aortic valve dysfunction [3]. The etiologies of these associations are unknown.

While multiple studies have demonstrated the high heritability of BAV, the underlying genetic causes remain poorly understood [4-7]. *NOTCH1* and *GATA5* are the only genes that have been linked to bicuspid aortic valve in humans, yet variants in these genes are present in only a minority of individuals with BAV [8-14]. Mice lacking *Gata5* have partially penetrant BAV of the R-NC subtype, but human studies have not yet demonstrated a specific association between *GATA5* variants and the R-NC subtype of BAV. Animal models of R-L BAV demonstrate excess fusion of the septal and parietal ridges of the outflow tract, whereas R-NC BAVs result from fusion of the septal ridge and posterior intercalated cushions [15]. These studies suggest that these two cusp fusion phenotypes may arise from distinct genetic perturbations in humans.

Despite tremendous advances in gene sequencing technology, the genetic etiology of many common human conditions, including BAV, remains poorly understood. Candidate gene studies have long been used to detect variants in individual genes; such studies are easy to perform but require selection of genes with a proposed role in the disease process of interest. Genome-wide association studies allow investigators to compare multiple individuals with a given condition and identify common variants in a non-candidate driven approach [16]. However, because genome-wide association studies are predicated upon the common disease-common variant hypothesis, this approach is not ideal for the study of rare variants, particularly in complex conditions in which rare variants at multiple loci may be needed to produce a clinically recognizable phenotype [17,18].

Next-generation sequencing (NGS) provides an opportunity for rapid, high-throughput sequencing of entire patient genomes and may overcome the limitation of genome-wide association studies in exploring the role of rare variants in complex diseases [19]. Whole genome sequencing remains at this time a costly technology, thus limiting its application to the sequencing of large cohorts of patients. It also produces a vast amount of data necessitating extensive bioinformatics processing. One option to overcome this issue is the design of targeted capture kits that allow for the rapid and accurate sequencing of only the genetic regions of interest. The two most common approaches to this technique have distinct limitations. Sequencing of a targeted set of genes can be done on individual samples, but this approach is very costly in larger cohorts. Alternatively, sequencing can be performed on pools of individual samples, wherein each sample is labeled with a unique genetic “barcode”; this approach is cost saving, but is quite labor intensive [20]. Combinatorial pooling schemes, wherein individuals are sampled in multiple pools, have been utilized to overcome these pitfalls and still permit identification of the individual sample contributing a given rare variant [21,22].

Here, we present an approach using combinatorial pooling and targeted multi-gene sequencing to study a well-phenotyped cohort of individuals with BAV. We hypothesize that rare variants will be identified amongst a large proportion of the candidate genes, that multiple rare variants will be found in individual probands, and that such variants will segregate by cusp fusion phenotype.

## Results

### Identification of sequence variants

We studied a previously described cohort of 78 patients with echocardiogram-identified BAV [8]. Using a targeted capture approach, we sequenced 97 candidate genes selected by reviewing the literature for genes relevant to heart valve development.

The average depth of coverage for the targeted regions was 268X. Greater than 50X coverage was obtained for 99.04% of the bases sequenced (range: 94.19-99.62), with greater than 100X for 96.11% of bases covered. The percentage of sequencing on target was 71.81%.

Targeted capture identified 42 rare, non-synonymous, exonic variants involving 35 of the candidate genes (Additional file 1: Table S1). Among these variants, *in-silico* analysis classified 33 of these 42 variants as putative disease-causing changes; Sanger sequencing did not validate two of these 33 variants. The remaining 31 changes were identified in 16 individuals and involved 28 genes (Table 1). Each variant was identified in only one proband. There were no significant differences in variant burden among BAV fusion phenotypes or isolated BAV versus BAV-CoA, with p = 0.78 and p = 0.77, respectively (Additional file 2: Table S2). Only 2 of these variants (rs72541816 at *APC* and rs116164480 at *GATA5*) were *de novo* changes not present in either parent of the affected probands. These two variants were identified in the same individual with a family history of coarctation of the aorta. Of the 16 individuals in whom putative disease-causing variants were identified, two had variants in genes previously known to be involved in human BAV (*NOTCH1, GATA5*), one of whom we previously described [8]. Four of these 16 individuals had a family history of a left ventricular outflow tract malformation.

**Table 1:** **Rare, non-synonymous, exonic variants in BAV cohort predicted damaging by *in-silico *** **analysis, confirmed by Sanger sequencing**

**Gene name**	**Nucleotide change**	**Amino acid change**	**De novo**	**SIFT**	**PP2**	**EA EVS**	**All EVS**	**1000G MAF**	**dbSNP137 ID**
**APC**	c.C7862G	p.S2621C	yes	0.03	0.641	0.005	0.003	0.058	rs72541816
**AXIN1**	c.G2522A	p.R841Q	no	0.4	1	0.012	0.008	0.01	rs34015754
**AXIN2**	c.C2051T	p.A684V	no	0.01	0.95	0.002	0.001	0	rs138287857
**FLT1**	c.C3092G	p.S1031C	no	0	1	0	0	0	N/A
**GATA4**	c.G1310C	p.G437A	no	0	0.787	0	0	0	N/A
**GATA5**	c.T698C	p.L233P	yes	0.05	0.723	0.001	0.001	0.003	rs116164480
**GLI1**	c.G3142A	p.D1048N	no	0	1	0	0	0	N/A
**JAG1**	c.G2810A	p.R937Q	no	0.47	0.093	0.002	0.001	0.001	rs145895196
**MCTP2**	c.C1634T	p.T545M	unknown	0	1	0	0	0	N/A
**MCTP2**	c.C2539T	p.L847F	no	0	1	0.0002	0.0002	0	rs150149342
**MSX1**	c.A581G	p.K194R	no	0	0.878	0.0003	0.0002	0	rs149092063
**NFATC1**	c.C230T	p.P77L	no	0	0.972	0	0	0	rs143045693
**NFATC1**	c.G628A	p.V210M	no	0.04	1	0	0	0	rs62096875
**NOS1**	c.G1975A	p.A659T	no	0	1	0	0	0	N/A
**NOTCH1**	c.C6481T	p.P2161S	unknown	0.02	0.975	0.0002	0.0002	0.001	rs201518848
**NOTCH2**	c.G6363C	p.K2121N	no	0.09	0.964	0.0008	0.0005	0	rs144047610
**NOTCH3**	c.A509G	p.H170R	no	0.01	0.974	0.002	0.001	0.001	rs147373451
**PAX6**	c.G1225A	p.G409R	no	0	1	0	0	0	N/A
**PIGF**	c.A370G	p.T124A	no	0.27	0.711	0.002	0.002	0.001	rs139098189
**PPP3CA**	c.C334T	p.R112C	no	0	1	0	0	0	N/A
**PTCH1**	c.G3487A	p.G1163S	no	0.06	1	0.0006	0.0006	0.001	rs113663584
**PTCH2**	c.C3139T	p.R1047W	no	0	0.998	0	0	0	N/A
**SLC35B2**	c.A1105G	p.I369V	no	0.04	0.891	0	0.00008	0	N/A
**SNAI3**	c.C488T	p.T163M	no	0.02	0.752	0	0	0.001	rs202205064
**SOX9**	c.G817C	p.V273L	no	0	0.719	0	0	0	rs201477430
**TBX5**	c.C1115T	p.S372L	no	0.65	0.861	0.0003	0.0002	0.001	rs143068551
**TBX5**	c.G787A	p.V263M	no	0.41	0.995	0	0.004	0.006	rs147405081
**VEGFB**	c.C286G	p.Q96E	no	0	0.596	0.002	0.002	0.002	rs111555072
**VEGFC**	c.A140T	p.E47V	no	0.01	0.985	0.005	0.004	0	rs55728985
**WNT4**	c.C129A	p.C43X	no	STOP	STOP	0	0	0	N/A
**ZNF236**	c.C4628T	p.P1543L	no	0.03	0.943	0	0	0	N/A

PP2; Polyphen 2.

EA, European American.

EVS, Exome Variant Server.

1000G, 1000 Genomes.

### Pathway analysis

Pathway analysis was performed using the Database for Annotation, Visualization and Integrated Discovery (DAVID). Pathway analysis was used to draw comparisons between the background of only those genes included in the targeted capture and the subset of genes in which rare, non-synonymous exonic variants predicted damaging by *in silico* analysis were identified. The pathway analysis revealed significant enrichment in genes involved in the WNT signaling pathway (p = 0.035).

### Pooling design validation

All samples in the cohort underwent Sanger sequencing of the coding regions of *GATA5* as previously reported by our group, used here as a test of the pooling design as well as the sensitivity and specificity of the variant calling algorithm. Four rare variants in *GATA5* (each present in 1/78 individuals) were discovered by Sanger sequencing, of which three were identified by NGS [8]. All of the rare *GATA5* variants identified by our NGS pooling design were attributed to the correct individual as confirmed by Sanger sequencing.

Sanger sequencing of *GATA5* found a variant, p.Q3R, in one individual that was not identified through the pooling design [8]. No pool had this variant above our cut-off threshold of 2.5% (four pools had allele frequencies over 1% with a range of 1.06-1.36%). Coverage of this base was good, with average read depth of 460X.

## Discussion

This NGS design utilizing targeted sequencing of pooled BAV patient samples identified 33 rare, non-synonymous exonic variants predicted damaging by *in silico* analysis. Traditional Sanger sequencing methods confirmed 31 of these 33 changes (94%). Analysis of the *GATA5* comparison dataset indicated that the pooling scheme allowed for accurate subject identification. This investigation identified rare variants in 26 genes not previously known to be involved in human BAV; such variants are considered hypothesis-generating and merit further testing in replication cohorts.

Animal models of BAV suggest a possible genotype-phenotype correlation related to cusp fusion phenotypes. However, our data does not support such a correlation in regards to cusp fusion, nor was there a correlation for isolated BAV versus BAV associated with coarctation of the aorta. Sample size and low incidence of familial BAV may limit our ability to detect such an association, but other groups have had similar findings. Rare, non-synonymous exonic variants in *GATA5* have not been shown to correlate with cusp fusion [8,13]. Investigations of familial BAV in large cohorts have demonstrated that cusp fusion morphologies were inherited interchangeably within families [23,24]. Taken together, these studies suggest that differing BAV phenotypes may derive from a common genetic pathway influenced by downstream modifying elements. Thorough testing of genotype-phenotype correlations would require larger cohorts with significant representation of cusp fusion phenotypes, associated congenital cardiac malformations, aortopathy, and aortic valve insufficiency/stenosis.

Prior to this study, only *GATA5* and *NOTCH1* variants had been associated with isolated human BAV. Our data identified variants in 26 additional genes not previously identified in human BAV patients. Interestingly, all of these variants are reported in less than 1% of the Exome Variant Server controls and half are absent in this control population. Nonetheless, only 2 of the 31 putative disease-causing changes confirmed by traditional sequencing methods were *de novo*, in that they were not identified in either parent of the affected proband. We speculate that these 31 variants may be susceptibility alleles, with additional factors (genetic or environmental) required for full phenotype expression [25]. Our finding of multiple variants in the same proband further supports this hypothesis. Among the 16 individuals in whom putative disease-causing variants were identified, the mean variant burden was 1.8 with a range of 1 to 5.

Pathway analysis provides an opportunity to ascribe further meaning to the large number of candidate genes that may be identified in high-throughput approaches such as the one described here. Bioinformatics analysis via DAVID identified significant enrichment of WNT pathway genes including *WNT4*, *PPP3CA*, *NFATC1*, *APC*, *AXIN1* and *AXIN 2*. DAVID pathway analysis can compare a subset of variants to any background of an investigator’s choosing; by utilizing a background of only the genes included in the targeted capture as opposed to the whole genome, the pathway analysis is not biased by overrepresentation of WNT pathway genes in the targeted capture design. WNT pathway genes display variable expression at various stages in valvulogenesis and have also been implicated in calcific valvular degeneration [26,27]. Coupling of NGS with pathway analysis allows for the development of more targeted sequencing approaches for subsequent studies. Further investigation into this and similar BAV cohorts could include an enhanced focus on the WNT signaling pathway. A more narrow scope of investigation would then facilitate advanced functional investigations of identified variants.

Several methods are now available for combining multiple individuals into a single sequencing run. Sample-specific indexing uses a short barcode sequence that is unique to each individual in a pool. This barcode is attached to the adapter sequence during library preparation. Commercially available kits now allow up to 96 individuals to be combined in a single run, with deconvolution allowing identification of the individual. Some problems remain in identifying correctly which sequence reads belong to the individual tagged, particularly if single (one end) indexing is used. The pooling method used here does not allow direct deconvolution, but it is not difficult to identify the individual possessing the identified variant. However, the pooling method offers the advantage of error mitigation through use of biological replicates, reducing the false positive rate due to the high frequency of sequencing errors in NGS [28]. Pooling will also overcome problems inherent in the indexing technique itself (including double indexing) that lead to sequencing errors [29].

More precise estimates of the pooling strategy false negative rates and investigation into the causes of these false negatives are necessary to improve the technique. The *GATA5* p.Q3R variant may have been missed for a variety of reasons including, but not limited to: error in DNA concentration measurement of the individual possessing the variant, volume measurement variability during pooling, or stochastic events during sequencing. One potential solution may be utilizing different DNA quantification methods for more accurate concentration prior to pooling. Additionally, a combinatorial design wherein each individual is represented in exactly three rather than two pools would potentially reduce false positive and negative rates.

A cost analysis of our approach showed significant savings. Targeted capture used in conjunction with the pooling scheme herein described resulted in a total sequencing cost of $15,950 for the entire 78 proband cohort. Targeted capture without pooling would have a total cost of $54,300 for a cohort of the same sample number, representing a cost savings of $39,350 from pooling alone. Moreover, assuming a cost of $1200 per sample for whole exome sequencing, the pooled and targeted approach would produce a relative cost saving of $77,650 for this cohort as compared to whole exome sequencing without pooling. Compared to whole genome sequencing without pooling (assumed to cost $5950 per sample), the pooled and targeted technique would realize a savings of $448,150.

## Conclusions

This unique approach to targeted gene sequencing identified a large number of putative disease-causing variants in a cohort of patients with BAV, including variants in 26 genes not previously associated with human BAV. Pathway analysis supported a role for WNT pathway genes in human BAV. The data as a whole further underscore the complex, polygenic nature of BAV. This technique provides a method for sample multiplexing that lowers costs and reduces sequencing errors.

## Methods

### Study population

The study cohort, previously described by our group, included 78 unrelated individuals (59 male, 19 female) with BAV [8]. Subjects were prospectively recruited from June 2004 to June 2011 as part of a larger study involving genetic testing in patients with congenital left ventricular tract outflow defects. Informed consent was obtained from study subjects or parents of subjects less than 18 years of age (assent was obtained from subjects 9–17 years of age) under protocols approved by the Institutional Review Board (IRB) at Nationwide Children’s Hospital. Subjects with known chromosomal abnormalities were excluded from the analysis. The majority of individuals were of Caucasian ethnicity, with 1 African-American, 1 Asian, and 3 Hispanic individuals. Each subject had undergone clinical echocardiography with images sufficient to identify associated cardiac malformations and aortic valve cusp fusion morphology (Table 2). Fifty of the 78 subjects (64%) had isolated BAV while the remainder had BAV-CoA. Forty-six subjects (59%) had R-L cusp fusion, 39% had R-NC fusion, and 2% had L-NC fusion. Eighteen of the 78 subjects had a family history of a left ventricular outflow tract defect. For the majority of subjects, parent samples were also obtained under the same IRB protocol. Genomic DNA was isolated from blood or saliva samples using the 5 PRIME DNA extraction kit (Thermo Fisher Scientific, Pittsburgh, PA).

**Table 2:** Cardiac phenotype of study population

	**BAV**	**BAV-CoA**	**Overall**
**R-L**	27(34.5%)	20(25.5%)	47(60%)
**R-NC**	22(28%)	7(9%)	30(38.5%)
**L-NC**	1(1%)	1(1%)	2(2.5%)
**Overall**	50(64%)	28(36%)	

BAV, bicuspid aortic valve (isolated).

BAV-CoA, bicuspid aortic valve with coarctation of the aorta.

R-L, fusion of right coronary cusp and left coronary cusp.

R-NC, fusion of right coronary cusp and non-coronary cusp.

L-NC, fusion of left coronary cusp and non-coronary cusp.

### Pooling scheme

Proband genomic DNA was combined into 19 unique pools each representing 9 or 10 individuals. The pools were constructed using overlapping design such that each individual was represented in exactly two pools, and a given rare variant could be uniquely attributed to a single individual on the basis of which two pools contained the variant. Individual genomic DNA samples were quantified by Nanodrop (Thermo Fisher Scientific), diluted to a concentration of 200 ng/microliter, and then requantified by Qubit fluorometer (Invitrogen Life Technologies, Carlsbad, CA). Quality of the DNA was assessed by SYBR Gold agarose gel (Life Technologies). Samples were then pooled, with the total amount of DNA for each pool consisting of 5 micrograms in 50 microliters (i.e. 500 ng per sample for a pool of 10 individuals and 550 ng per sample for a pool of 9 individuals).

### Targeted capture

A custom, targeted gene capture was designed using the Agilent SureSelect Target Enrichment kit (Table 3). Candidate genes were selected on the basis of relevance to cardiac development and/or congenital heart defects in humans and animal models. Reference sequences were obtained from the Ensembl database. Probes were designed using paired, double-end, 75 base pair reads with centered design and 2x tiling frequency. A total of 97 candidate genes were probed using a whole gene interval approach, representing 7.6 Mb of DNA. Analysis was subsequently confined to exonic regions.

**Table 3:** Targeted capture gene list

**Ensembl gene ID**	**Gene name**	**Chromosome**	**Gene start (bp)**	**Gene end (bp)**	**Size**
**ENSG00000107796**	ACTA2	10	90694831	90751147	56316
**ENSG00000115170**	ACVR1	2	158592958	158732374	139416
**ENSG00000134982**	APC	5	112043195	112181936	138741
**ENSG00000081181**	ARG2	14	68086515	68118437	31922
**ENSG00000103126**	AXIN1	16	337440	402673	65233
**ENSG00000168646**	AXIN2	17	63524681	63557765	33084
**ENSG00000149541**	B3GAT3	11	62382768	62389647	6879
**ENSG00000242252**	BGLAP	1	156211753	156213112	1359
**ENSG00000125845**	BMP2	20	6748311	6760910	12599
**ENSG00000125378**	BMP4	14	54416454	54425479	9025
**ENSG00000107779**	BMPR1A	10	88516396	88684945	168549
**ENSG00000138696**	BMPR1B	4	95679119	96079599	400480
**ENSG00000204217**	BMPR2	2	203241659	203432474	190815
**ENSG00000134072**	CAMK1	3	9799026	9811676	12650
**ENSG00000105974**	CAV1	7	116164839	116201233	36394
**ENSG00000179776**	CDH5	16	66400533	66438686	38153
**ENSG00000132535**	DLG4	17	7093209	7123369	30160
**ENSG00000198719**	DLL1	6	170591294	170599561	8267
**ENSG00000090932**	DLL3	19	39989557	39999118	9561
**ENSG00000128917**	DLL4	15	41221538	41231237	9699
**ENSG00000106991**	ENG	9	130577291	130617035	39744
**ENSG00000138685**	FGF2	4	123747863	123819391	71528
**ENSG00000107831**	FGF8	10	103530081	103535827	5746
**ENSG00000102755**	FLT1	13	28874489	29069265	194776
**ENSG00000136574**	GATA4	8	11534468	11617511	83043
**ENSG00000130700**	GATA5	20	61038553	61051026	12473
**ENSG00000141448**	GATA6	18	19749404	19782491	33087
**ENSG00000111087**	GLI1	12	57853918	57866045	12127
**ENSG00000074047**	GLI2	2	121493199	121750229	257030
**ENSG00000106571**	GLI3	7	42000548	42277469	276921
**ENSG00000105464**	GRIN2D	19	48898132	48948187	50055
**ENSG00000164116**	GUCY1A3	4	156587863	156653501	65638
**ENSG00000061918**	GUCY1B3	4	156680144	156728743	48599
**ENSG00000164683**	HEY1	8	80676245	80680098	3853
**ENSG00000135547**	HEY2	6	126068810	126082415	13605
**ENSG00000163909**	HEYL	1	40089825	40105617	15792
**ENSG00000080824**	HSP90AA1	14	102547106	102606036	58930
**ENSG00000096384**	HSP90AB1	6	44214824	44221620	6796
**ENSG00000166598**	HSP90B1	12	104323885	104347423	23538
**ENSG00000101384**	JAG1	20	10618332	10654608	36276
**ENSG00000184916**	JAG2	14	105607318	105635161	27843
**ENSG00000123700**	KCNJ2	17	68164814	68176160	11346
**ENSG00000127528**	KLF2	19	16435651	16438337	2686
**ENSG00000140563**	MCTP2	15	94774767	95023632	248865
**ENSG00000087245**	MMP2	16	55423612	55540603	116991
**ENSG00000163132**	MSX1	4	4861393	4865663	4270
**ENSG00000120149**	MSX2	5	174151536	174158144	6608
**ENSG00000131196**	NFATC1	18	77155772	77289325	133553
**ENSG00000183072**	NKX2-5	5	172659112	172662360	3248
**ENSG00000089250**	NOS1	12	117645947	117889975	244028
**ENSG00000007171**	NOS2	17	26083792	26127525	43733
**ENSG00000164867**	NOS3	7	150688083	150711676	23593
**ENSG00000148400**	NOTCH1	9	139388896	139440314	51418
**ENSG00000134250**	NOTCH2	1	120454176	120612240	158064
**ENSG00000074181**	NOTCH3	19	15270445	15311792	41347
**ENSG00000204301**	NOTCH4	6	32162620	32191844	29224
**ENSG00000151665**	PIGF	2	46808076	46844258	36182
**ENSG00000076356**	PLXNA2	1	208195587	208417665	222078
**ENSG00000132170**	PPARG	3	12328867	12475855	146988
**ENSG00000138814**	PPP3CA	4	101944566	102269435	324869
**ENSG00000188191**	PRKAR1B	7	588834	767287	178453
**ENSG00000154229**	PRKCA	17	64298754	64806861	508107
**ENSG00000080815**	PSEN1	14	73603126	73690399	87273
**ENSG00000143801**	PSEN2	1	227057885	227083806	25921
**ENSG00000185920**	PTCH1	9	98205262	98279339	74077
**ENSG00000117425**	PTCH2	1	45285516	45308735	23219
**ENSG00000131759**	RARA	17	38465444	38513094	47650
**ENSG00000077092**	RARB	3	25215823	25639423	423600
**ENSG00000172819**	RARG	12	53604354	53626764	22410
**ENSG00000124813**	RUNX2	6	45295894	45632086	336192
**ENSG00000186350**	RXRA	9	137208944	137332431	123487
**ENSG00000204231**	RXRB	6	33161365	33168630	7265
**ENSG00000143171**	RXRG	1	165370159	165414433	44274
**ENSG00000162572**	SCNN1D	1	1214447	1227409	12962
**ENSG00000075223**	SEMA3C	7	80371854	80551675	179821
**ENSG00000164690**	SHH	7	155592680	155604967	12287
**ENSG00000128602**	SMO	7	128828713	128853386	24673
**ENSG00000124216**	SNAI1	20	48599536	48605423	5887
**ENSG00000019549**	SNAI2	8	49830249	49834299	4050
**ENSG00000185669**	SNAI3	16	88744090	88752901	8811
**ENSG00000125398**	SOX9	17	70117161	70122561	5400
**ENSG00000184058**	TBX1	22	19744226	19771116	26890
**ENSG00000121068**	TBX2	17	59477257	59486827	9570
**ENSG00000164532**	TBX20	7	35242042	35293758	51716
**ENSG00000089225**	TBX5	12	114791736	114846247	54511
**ENSG00000105329**	TGFB1	19	41836813	41859831	23018
**ENSG00000106799**	TGFBR1	9	101866320	101916474	50154
**ENSG00000163513**	TGFBR2	3	30647994	30735634	87640
**ENSG00000122691**	TWIST1	7	19060614	19157295	96681
**ENSG00000070010**	UFD1L	22	19437464	19466738	29274
**ENSG00000112715**	VEGFA	6	43737921	43754224	16303
**ENSG00000173511**	VEGFB	11	64002010	64006259	4249
**ENSG00000150630**	VEGFC	4	177604689	177713881	109192
**ENSG00000105989**	WNT2	7	116916685	116963343	46658
**ENSG00000162552**	WNT4	1	22446461	22470462	24001
**ENSG00000184937**	WT1	11	32409321	32457176	47855
**ENSG00000130856**	ZNF236	18	74534563	74682683	148120
				CAPTURE SIZE	7567444

### Sequencing

Sequencing of the pooled target captured proband genomic DNA was performed on the Illumina HiSeq 2000. Variants considered potentially pathogenic identified by NGS were subsequently confirmed by Sanger sequencing. Where available, parent samples were also sequenced for these potentially pathogenic variants. Sequencing primers are available upon request.

### Bioinformatics algorithms

Bioinformatics analysis was performed using Churchill, our laboratory’s pipeline for the discovery of human genetic variation. Churchill utilizes the Burrows Wheeler Aligner (BWA) for the alignment of sequence data to the reference genome, hg19. Further refinement steps were performed on the aligned sequence data using Genome Analysis ToolKit (GATK) following the Broad Institute’s guidelines for best practices (https://www.broadinstitute.org/gatk/guide/best-practices). We utilized the GATK’s (version 2.4-9) UnifiedGenotyper (UG) to call variants in the pooled samples. In order to properly handle the pooled data, we amended the recommended UG settings by including the –sample_ploidy configuration parameter and giving it a value of 20, reflecting the potential for 20 individual alleles in a pooled sample of 10 individuals. The threshold for calling was set to 2.5% alternate allele frequency on the basis of the pooling scheme.

### In-silico analysis

Rare, non-synonymous, exonic variants were analyzed using the Polyphen 2 and SIFT algorithms. Reference populations from the 1000 Genomes Project and Exome Variant Server were utilized as control populations [30,31]. Pathway analysis was performed using the Database for Annotation, Visualization and Integrated Discovery (DAVID) with cutoffs of p-value less than 0.05 [32,33].

### Availability of supporting data

This project has been registered with the National Center for Biotechnology Information (NCBI) BioProject database, identifier PRJNA260036, and can be accessed at: http://www.ncbi.nlm.nih.gov/bioproject/260036.

Supporting sequence data for this project has been deposited with the NCBI Sequence Read Archive. The study accession is SRP045998, available at the following link: http://www.ncbi.nlm.nih.gov/sra/?term=SRP045998 Biosample IDs for the pools, with their corresponding URLs are:

3015266: http://www.ncbi.nlm.nih.gov/biosample/3015266

3015267: http://www.ncbi.nlm.nih.gov/biosample/3015267

3015268: http://www.ncbi.nlm.nih.gov/biosample/3015268

3015269: http://www.ncbi.nlm.nih.gov/biosample/3015269

3015270: http://www.ncbi.nlm.nih.gov/biosample/3015270

3015271: http://www.ncbi.nlm.nih.gov/biosample/3015271

3015272: http://www.ncbi.nlm.nih.gov/biosample/3015272

3015273: http://www.ncbi.nlm.nih.gov/biosample/3015273

3015274: http://www.ncbi.nlm.nih.gov/biosample/3015274

3015275: http://www.ncbi.nlm.nih.gov/biosample/3015275

3015276: http://www.ncbi.nlm.nih.gov/biosample/3015276

3015277: http://www.ncbi.nlm.nih.gov/biosample/3015277

3015278: http://www.ncbi.nlm.nih.gov/biosample/3015278

3015279: http://www.ncbi.nlm.nih.gov/biosample/3015279

3015280: http://www.ncbi.nlm.nih.gov/biosample/3015280

3015281: http://www.ncbi.nlm.nih.gov/biosample/3015281

3015282: http://www.ncbi.nlm.nih.gov/biosample/3015282

3015283: http://www.ncbi.nlm.nih.gov/biosample/3015283

3015284: http://www.ncbi.nlm.nih.gov/biosample/3015284

3015285: http://www.ncbi.nlm.nih.gov/biosample/3015285

## Abbreviations

BAV: Bicuspid aortic valve; BAV-CoA: Bicuspid aortic valve associated with coarctation of the aorta; R-L: Right coronary and left coronary; R-NC: Right coronary and non-coronary; NGS: Next-generation sequencing; DAVID: Database for annotation, visualization and integrated discovery; GATK: Genome analysis toolkit; UG: UnifiedGenotyper.

## Competing interests

The authors declare that they have no competing interests.

## Authors’ contributions

EB participated in study conception and design, sample preparation, statistical analysis, and drafted the manuscript. GZ participated in sample preparation and sequencing. PW, DN, and DC participated in sequencing, bioinformatics processing, and data interpretation. SFB participated in study conception and study recruitment. VG and KM participated in study conception and design, study coordination, and helped to draft the manuscript. All authors read and approved the final manuscript.

## Pre-publication history

The pre-publication history for this paper can be accessed here:

http://www.biomedcentral.com/1755-8794/7/56/prepub

## Supplementary Material

Additional file 1: Table S1 Rare, non-synonymous, exonic variants in BAV cohort. Click here for file

Additional file 2: Table S2 Clinical characteristics of probands with rare, non-synonymous, exonic variants predicted damaging by *in-silico* analysis and confirmed by Sanger sequencing. Click here for file
