# Supplementary material for: Use of a targeted, combinatorial next-generation sequencing approach for the study of bicuspid aortic valve
Source: BMC Med Genomics. 2014 Sep 26;7:56. doi: 10.1186/1755-8794-7-56 (PMC4181662; doi:10.1186/1755-8794-7-56)
Supplement: Additional file 1: Table S1 — Rare, non-synonymous, exonic variants in BAV cohort. [file 1755-8794-7-56-S1.doc]

Supplementary Table 1. Rare, non-synonymous, exonic variants in BAV cohort

| **Gene Name** | **Nucleotide Change** | **Amino Acid Change** | **dbSNP137 ID** |
| --- | --- | --- | --- |
| **APC** | c.C7862G | p.S2621C | rs72541816 |
| **AXIN1** | c.G2522A | p.R841Q | rs34015754 |
| **AXIN2** | c.C2239T | p.H747Y | rs143571197 |
| **AXIN2** | c.C2051T | p.A684V | rs138287857 |
| **FLT1** | c.C3092G | p.S1031C | N/A |
| **GATA4** | c.G1310C | p.G437A | N/A |
| **GATA5** | c.T698C | p.L233P | rs116164480 |
| **GLI1** | c.C652T | p.R218W | N/A |
| **GLI1** | c.G3142A | p.D1048N | N/A |
| **HEATR2** | c.C550A | p.R184S | N/A |
| **JAG1** | c.G2810A | p.R937Q | rs145895196 |
| **JAG2** | c.C3467T | p.P1156L | rs143438617 |
| **MCTP2** | c.C1634T | p.T545M | N/A |
| **MCTP2** | c.C2539T | p.L847F | rs150149342 |
| **MMP2** | c.C1758A | p.D586E | N/A |
| **MSX1** | c.A581G | p.K194R | rs149092063 |
| **NFATC1** | c.C230T | p.P77L | rs143045693 |
| **NFATC1** | c.G628A | p.V210M | rs62096875 |
| **NOS1** | c.G1975A | p.A659T | N/A |
| **NOS1** | c.A1855T | p.M619L | rs79487279 |
| **NOS2** | c.G2913C | p.E971D | rs145672724 |
| **NOTCH1** | c.C6481T | p.P2161S | rs201518848 |
| **NOTCH2** | c.G6363C | p.K2121N | rs144047610 |
| **NOTCH3** | c.A509G | p.H170R | rs147373451 |
| **OGG1** | c.G964A | p.D322N | rs3219014 |
| **PAX6** | c.G1225A | p.G409R | N/A |
| **PIGF** | c.A370G | p.T124A | rs139098189 |
| **PPP3CA** | c.C334T | p.R112C | N/A |
| **PTCH1** | c.G3487A | p.G1163S | rs113663584 |
| **PTCH2** | c.C3139T | p.R1047W | N/A |
| **SLC35B2** | c.A1105G | p.I369V | N/A |
| **SMO** | c.G808A | p.V270I | rs111694017 |
| **SNAI3** | c.A83G | p.N28S | rs201702547 |
| **SNAI3** | c.C488T | p.T163M | rs202205064 |
| **SOX9** | c.G817C | p.V273L | rs201477430 |
| **TBX5** | c.C1115T | p.S372L | rs143068551 |
| **TBX5** | c.G787A | p.V263M | rs147405081 |
| **TGFBR1** | c.A214T | p.I72L | rs111513627 |
| **VEGFB** | c.C286G | p.Q96E | rs111555072 |
| **VEGFC** | c.A140T | p.E47V | rs55728985 |
| **WNT4** | c.C129A | p.C43X | N/A |
| **ZNF236** | c.C4628T | p.P1543L | N/A |
