# Supplementary material for: Use of a targeted, combinatorial next-generation sequencing approach for the study of bicuspid aortic valve
Source: BMC Med Genomics. 2014 Sep 26;7:56. doi: 10.1186/1755-8794-7-56 (PMC4181662; doi:10.1186/1755-8794-7-56)
Supplement: Additional file 2: Table S2 — Clinical characteristics of probands with rare, non-synonymous, exonic variants predicted damaging by in-silico analysis and confirmed by Sanger sequencing. [file 1755-8794-7-56-S2.doc]

Supplemental Table 2. Clinical characteristics of probands with rare, non-synonymous, exonic variants predicted damaging by *in-silico* analysis and confirmed by Sanger sequencing

| **Sample Identifier** | **Gene Name** | **Nucleotide Change** | **Amino Acid Change** | **Cardiac Phenotype** | **Aortic valve phenotype** | **Gender** | **Ethnicity** | **Familial CHD** |
| --- | --- | --- | --- | --- | --- | --- | --- | --- |
| **a** | NOTCH2 | c.G6363C | p.K2121N | BAV | RNC | Male | Caucasian | BAV-AVS |
| **b** | GATA4 | c.G1310C | p.G437A | BAV-CoA | RL | Male | Caucasian | HLHS |
| **b** | PIGF | c.A370G | p.T124A | BAV-CoA | RL | Male | Caucasian | HLHS |
| **c** | APC | c.C7862G | p.S2621C | BAV-CoA | RNC | Male | Caucasian | CoA |
| **c** | GATA5 | c.T698C | p.L233P | BAV-CoA | RNC | Male | Caucasian | CoA |
| **d** | MSX1 | c.A581G | p.K194R | BAV | RL | Female | Caucasian | No |
| **e** | NOS1 | c.G1975A | p.A659T | BAV | RNC | Male | Caucasian | No |
| **e** | VEGFB | c.C286G | p.Q96E | BAV | RNC | Male | Caucasian | No |
| **f** | NOTCH3 | c.A509G | p.H170R | BAV | RL | Male | Caucasian | No |
| **f** | PPP3CA | c.C334T | p.R112C | BAV | RL | Male | Caucasian | No |
| **f** | TBX5 | c.C1115T | p.S372L | BAV | RL | Male | Caucasian | No |
| **g** | AXIN2 | c.C2051T | p.A684V | BAV | RL | Male | Caucasian | BAV |
| **h** | ZNF236 | c.C4628T | p.P1543L | BAV | RNC | Male | Caucasian | No |
| **i** | SNAI3 | c.C488T | p.T163M | BAV-CoA | RL | Female | Caucasian | BAV-AVS |
| **j** | MCTP2 | c.C1634T | p.T545M | BAV | RNC | Male | Caucasian | No |
| **j** | SOX9 | c.G817C | p.V273L | BAV | RNC | Male | Caucasian | No |
| **k** | FLT1 | c.C3092G | p.S1031C | BAV | RNC | Male | Caucasian | No |
| **k** | MCTP2 | c.C2539T | p.L847F | BAV | RNC | Male | Caucasian | No |
| **k** | PTCH1 | c.G3487A | p.G1163S | BAV | RNC | Male | Caucasian | No |
| **k** | PTCH2 | c.C3139T | p.R1047W | BAV | RNC | Male | Caucasian | No |
| **k** | WNT4 | c.C129A | p.C43X | BAV | RNC | Male | Caucasian | No |
| **l** | GLI1 | c.G3142A | p.D1048N | BAV | RL | Male | Hispanic | No |
| **m** | SLC35B2 | c.A1105G | p.I369V | BAV | RNC | Male | Caucasian | No |
| **n** | VEGFC | c.A140T | p.E47V | BAV-CoA | RL | Female | Caucasian | No |
| **o** | AXIN1 | c.G2522A | p.R841Q | BAV-CoA | RL | Male | Caucasian | No |
| **o** | NFATC1 | c.C230T | p.P77L | BAV-CoA | RL | Male | Caucasian | No |
| **o** | NFATC1 | c.G628A | p.V210M | BAV-CoA | RL | Male | Caucasian | No |
| **o** | TBX5 | c.G787A | p.V263M | BAV-CoA | RL | Male | Caucasian | No |
| **p** | JAG1 | c.G2810A | p.R937Q | BAV | RL | Male | Caucasian | No |
| **p** | NOTCH1 | c.C6481T | p.P2161S | BAV | RL | Male | Caucasian | No |
| **p** | PAX6 | c.G1225A | p.G409R | BAV | RL | Male | Caucasian | No |
